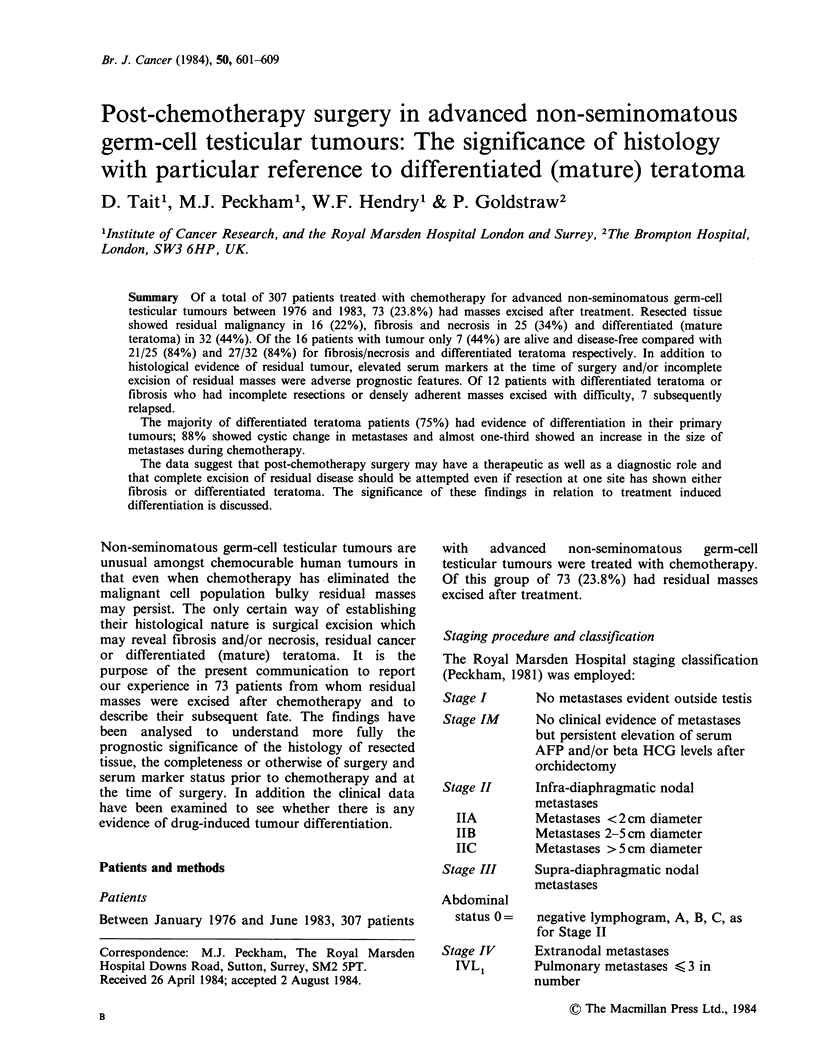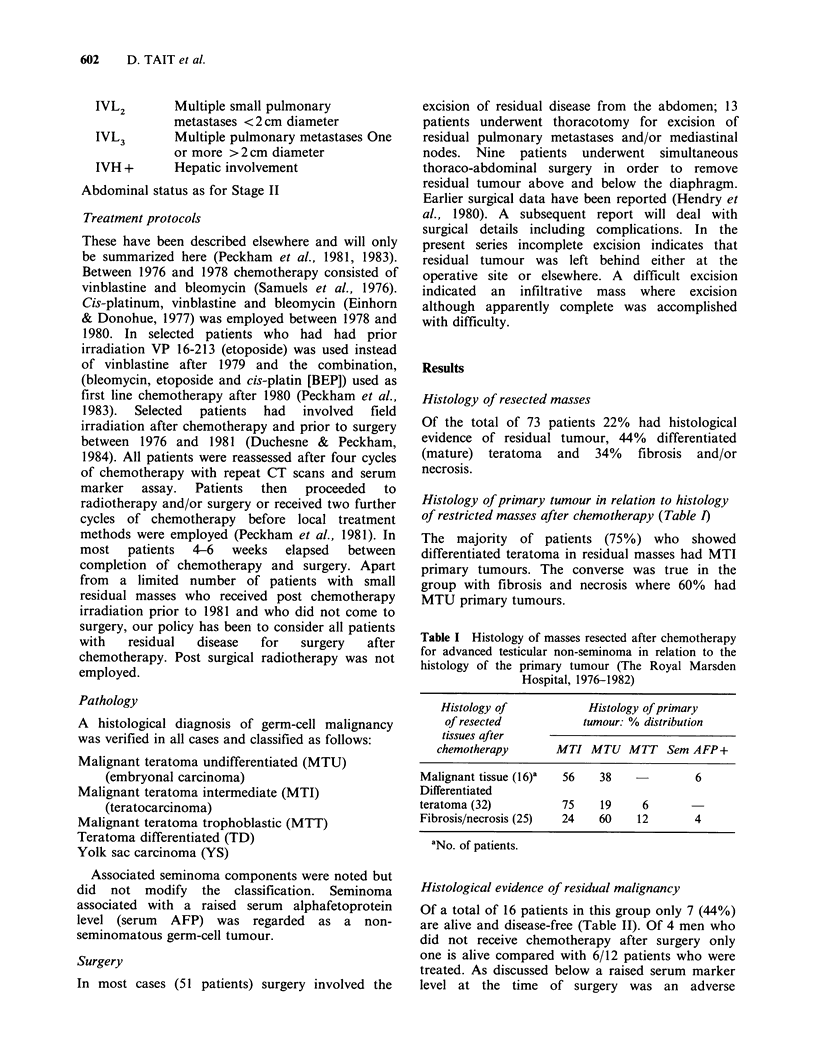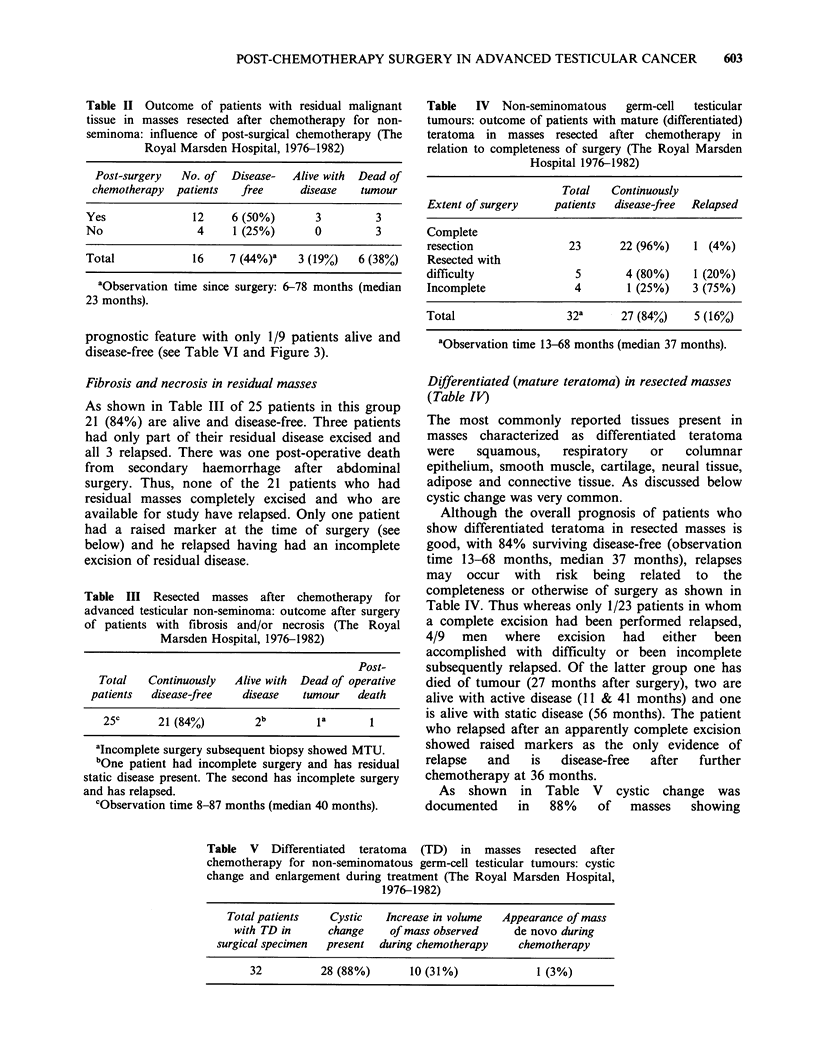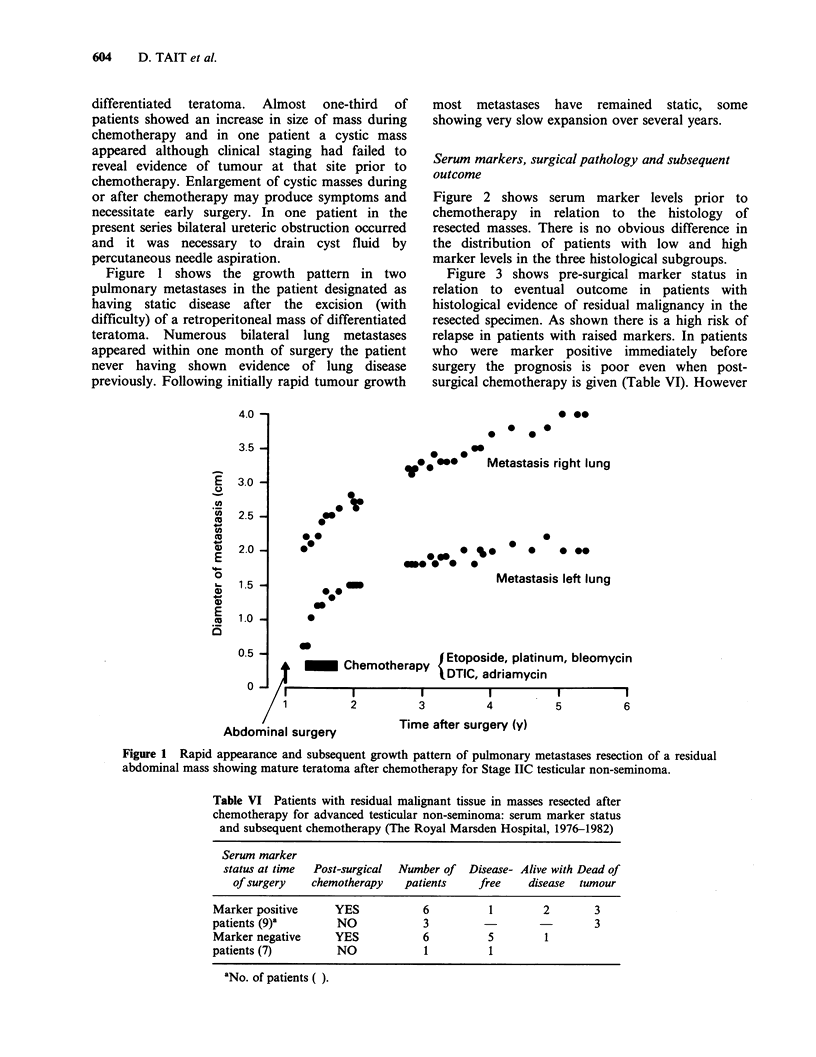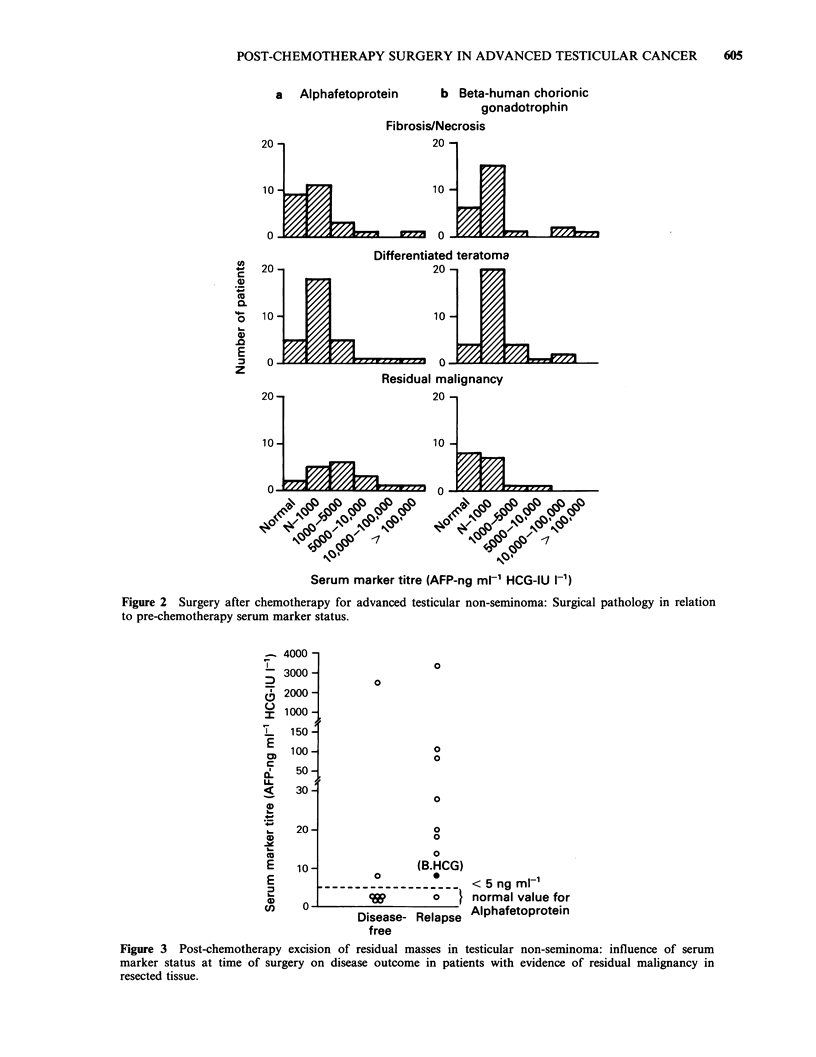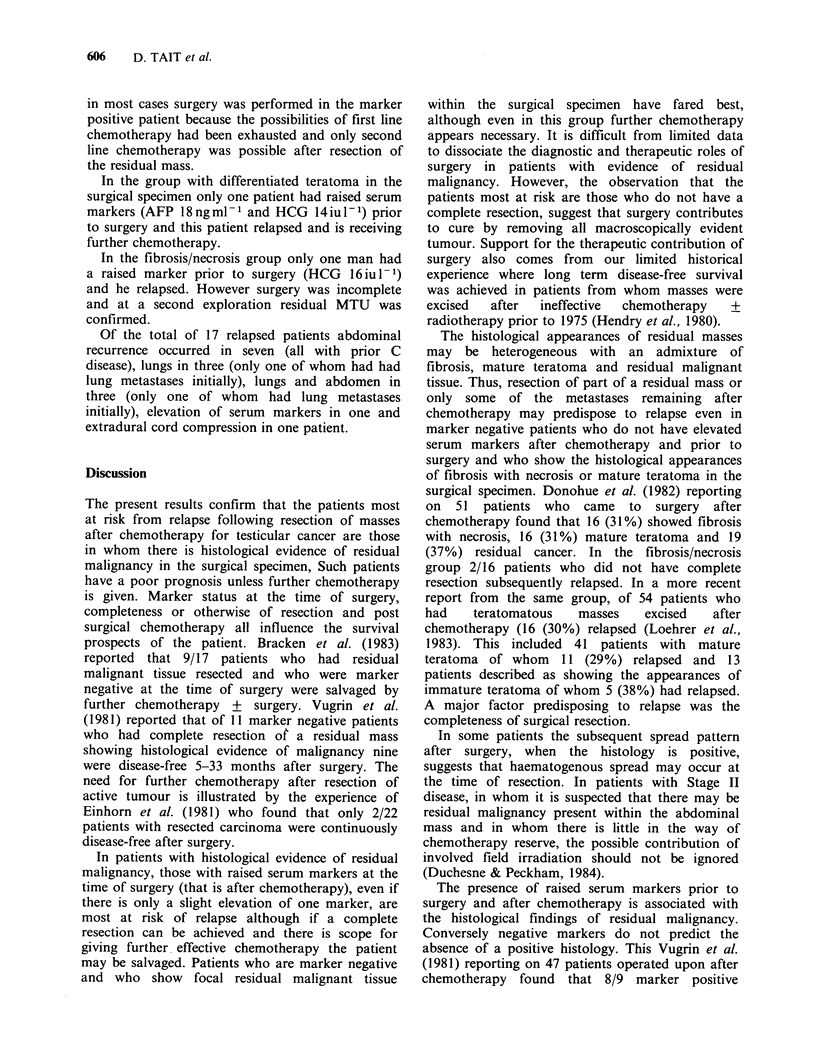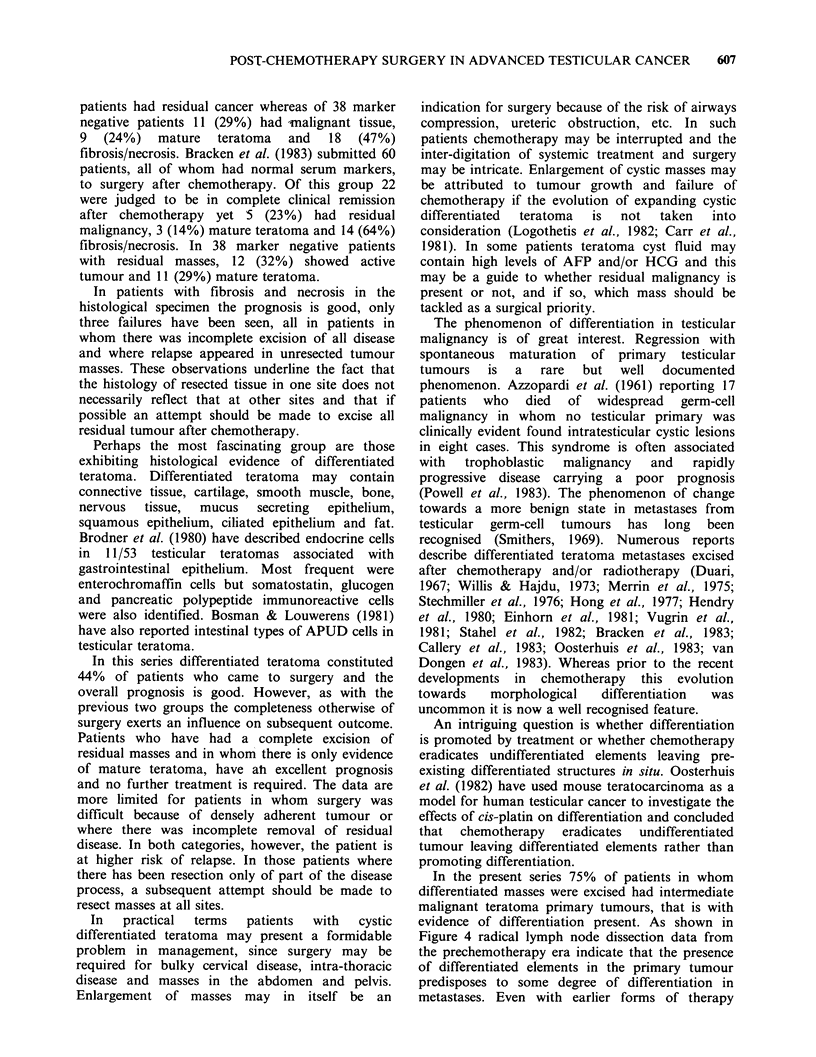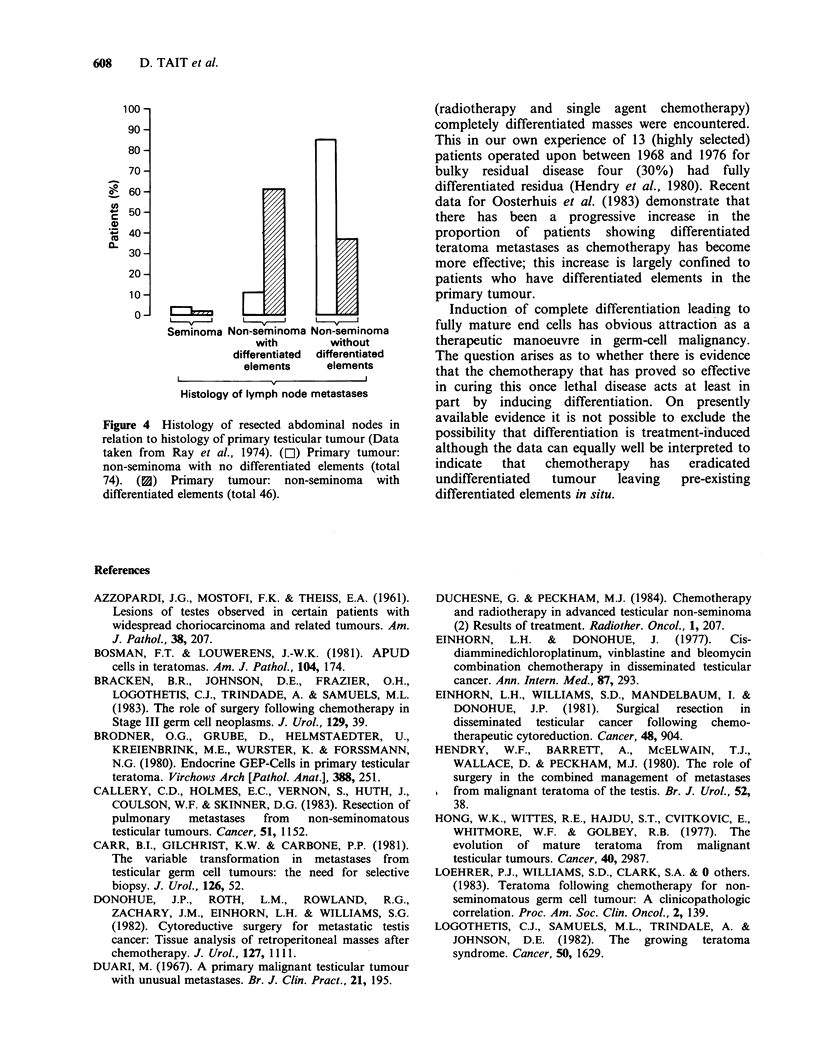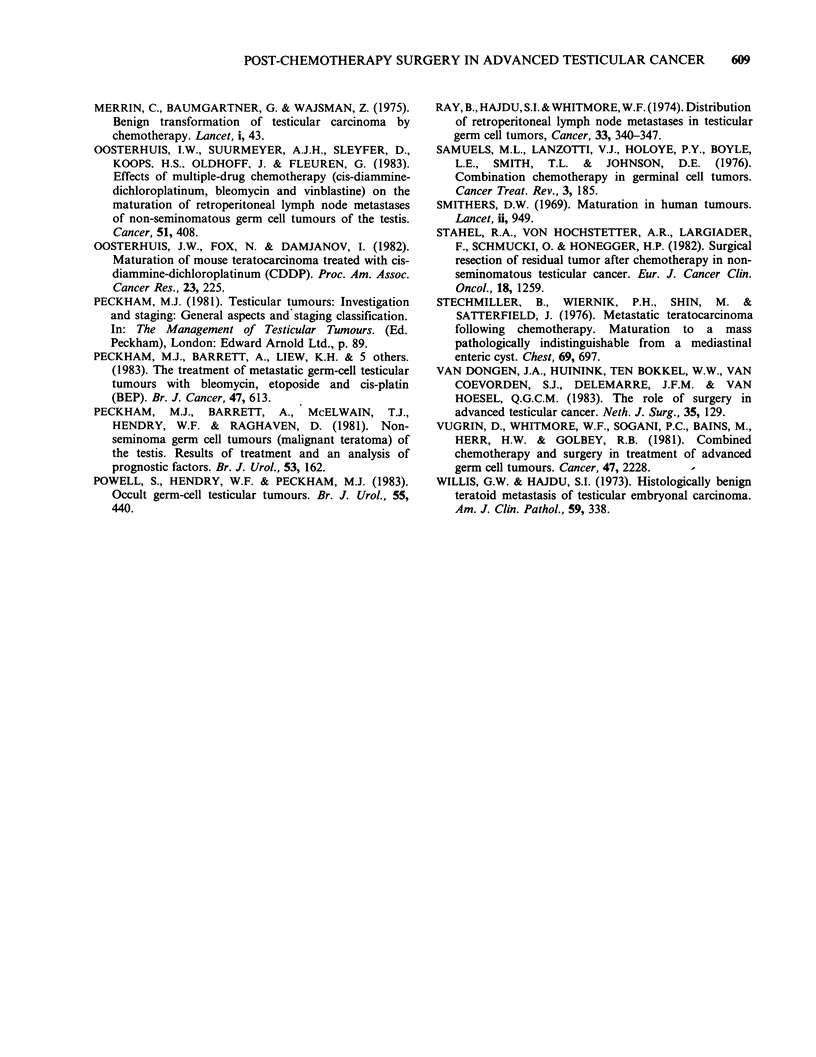# Post-chemotherapy surgery in advanced non-seminomatous germ-cell testicular tumours: the significance of histology with particular reference to differentiated (mature) teratoma.

**DOI:** 10.1038/bjc.1984.226

**Published:** 1984-11

**Authors:** D. Tait, M. J. Peckham, W. F. Hendry, P. Goldstraw

## Abstract

Of a total of 307 patients treated with chemotherapy for advanced non-seminomatous germ-cell testicular tumours between 1976 and 1983, 73 (23.8%) had masses excised after treatment. Resected tissue showed residual malignancy in 16 (22%), fibrosis and necrosis in 25 (34%) and differentiated (mature teratoma) in 32 (44%). Of the 16 patients with tumour only 7 (44%) are alive and disease-free compared with 21/25 (84%) and 27/32 (84%) for fibrosis/necrosis and differentiated teratoma respectively. In addition to histological evidence of residual tumour, elevated serum markers at the time of surgery and/or incomplete excision of residual masses were adverse prognostic features. Of 12 patients with differentiated teratoma or fibrosis who had incomplete resections or densely adherent masses excised with difficulty, 7 subsequently relapsed. The majority of differentiated teratoma patients (75%) had evidence of differentiation in their primary tumours; 88% showed cystic change in metastases and almost one-third showed an increase in the size of metastases during chemotherapy. The data suggest that post-chemotherapy surgery may have a therapeutic as well as a diagnostic role and that complete excision of residual disease should be attempted even if resection at one site has shown either fibrosis or differentiated teratoma. The significance of these findings in relation to treatment induced differentiation is discussed.


*Br. J. Cancer* (1984), **50**, 601–609

### Post-chemotherapy surgery in advanced non-seminomatous germ-cell testicular tumours: The significance of histology with particular reference to differentiated (mature) teratoma

D. Tait[1], M.J. Peckham[1], W.F. Hendry[1] & P. Goldstraw[2]

[1]*Institute of Cancer Research, and the Royal Marsden Hospital London and Surrey,* [2]*The Brompton Hospital, London, SW3 6HP, UK.*

**Summary** Of a total of 307 patients treated with chemotherapy for advanced non-seminomatous germ-cell testicular tumours between 1976 and 1983, 73 (23.8%) had masses excised after treatment. Resected tissue showed residual malignancy in 16 (22%), fibrosis and necrosis in 25 (34%) and differentiated (mature teratoma) in 32 (44%). Of the 16 patients with tumour only 7 (44%) are alive and disease-free compared with 21/25 (84%) and 27/32 (84%) for fibrosis/necrosis and differentiated teratoma respectively. In addition to histological evidence of residual tumour, elevated serum markers at the time of surgery and/or incomplete excision of residual masses were adverse prognostic features. Of 12 patients with differentiated teratoma or fibrosis who had incomplete resections or densely adherent masses excised with difficulty, 7 subsequently relapsed.

The majority of differentiated teratoma patients (75%) had evidence of differentiation in their primary tumours; 88% showed cystic change in metastases and almost one-third showed an increase in the size of metastases during chemotherapy.

The data suggest that post-chemotherapy surgery may have a therapeutic as well as a diagnostic role and that complete excision of residual disease should be attempted even if resection at one site has shown either fibrosis or differentiated teratoma. The significance of these findings in relation to treatment induced differentiation is discussed.

Non-seminomatous germ-cell testicular tumours are unusual amongst chemocurable human tumours in that even when chemotherapy has eliminated the malignant cell population bulky residual masses may persist. The only certain way of establishing their histological nature is surgical excision which may reveal fibrosis and/or necrosis, residual cancer or differentiated (mature) teratoma. It is the purpose of the present communication to report our experience in 73 patients from whom residual masses were excised after chemotherapy and to describe their subsequent fate. The findings have been analysed to understand more fully the prognostic significance of the histology of resected tissue, the completeness or otherwise of surgery and serum marker status prior to chemotherapy and at the time of surgery. In addition the clinical data have been examined to see whether there is any evidence of drug-induced tumour differentiation.

### Patients and methods

### *Patients*

Between January 1976 and June 1983, 307 patients with advanced non-seminomatous germ-cell testicular tumours were treated with chemotherapy. Of this group of 73 (23.8%) had residual masses excised after treatment.

Correspondence: M.J. Peckham, The Royal Marsden Hospital Downs Road, Sutton, Surrey, SM2 5PT.
Received 26 April 1984; accepted 2 August 1984.

### *Staging procedure and classification*

The Royal Marsden Hospital staging classification (Peckham, 1981) was employed:

| | |
|---|---|
| *Stage I* | No metastases evident outside testis |
| *Stage IM* | No clinical evidence of metastases but persistent elevation of serum AFP and/or beta HCG levels after orchidectomy |
| *Stage II* | Infra-diaphragmatic nodal metastases |
| IIA | Metastases <2 cm diameter |
| IIB | Metastases 2–5 cm diameter |
| IIC | Metastases >5 cm diameter |
| *Stage III* | Supra-diaphragmatic nodal metastases |
| Abdominal status 0= | negative lymphogram, A, B, C, as for Stage II |
| *Stage IV* | Extranodal metastases |
| $IVL_1$ | Pulmonary metastases ⩽3 in number |

© The Macmillan Press Ltd., 1984

| | |
|---|---|
| $IVL_2$ | Multiple small pulmonary metastases <2 cm diameter |
| $IVL_3$ | Multiple pulmonary metastases One or more >2 cm diameter |
| IVH+ | Hepatic involvement |

Abdominal status as for Stage II

*Treatment protocols*

These have been described elsewhere and will only be summarized here (Peckham *et al.*, 1981, 1983). Between 1976 and 1978 chemotherapy consisted of vinblastine and bleomycin (Samuels *et al.*, 1976). *Cis*-platinum, vinblastine and bleomycin (Einhorn & Donohue, 1977) was employed between 1978 and 1980. In selected patients who had had prior irradiation VP 16-213 (etoposide) was used instead of vinblastine after 1979 and the combination, (bleomycin, etoposide and *cis*-platin [BEP]) used as first line chemotherapy after 1980 (Peckham *et al.*, 1983). Selected patients had involved field irradiation after chemotherapy and prior to surgery between 1976 and 1981 (Duchesne & Peckham, 1984). All patients were reassessed after four cycles of chemotherapy with repeat CT scans and serum marker assay. Patients then proceeded to radiotherapy and/or surgery or received two further cycles of chemotherapy before local treatment methods were employed (Peckham *et al.*, 1981). In most patients 4–6 weeks elapsed between completion of chemotherapy and surgery. Apart from a limited number of patients with small residual masses who received post chemotherapy irradiation prior to 1981 and who did not come to surgery, our policy has been to consider all patients with residual disease for surgery after chemotherapy. Post surgical radiotherapy was not employed.

*Pathology*

A histological diagnosis of germ-cell malignancy was verified in all cases and classified as follows:

Malignant teratoma undifferentiated (MTU) (embryonal carcinoma)
Malignant teratoma intermediate (MTI) (teratocarcinoma)
Malignant teratoma trophoblastic (MTT)
Teratoma differentiated (TD)
Yolk sac carcinoma (YS)

Associated seminoma components were noted but did not modify the classification. Seminoma associated with a raised serum alphafetoprotein level (serum AFP) was regarded as a non-seminomatous germ-cell tumour.

*Surgery*

In most cases (51 patients) surgery involved the excision of residual disease from the abdomen; 13 patients underwent thoracotomy for excision of residual pulmonary metastases and/or mediastinal nodes. Nine patients underwent simultaneous thoraco-abdominal surgery in order to remove residual tumour above and below the diaphragm. Earlier surgical data have been reported (Hendry *et al.*, 1980). A subsequent report will deal with surgical details including complications. In the present series incomplete excision indicates that residual tumour was left behind either at the operative site or elsewhere. A difficult excision indicated an infiltrative mass where excision although apparently complete was accomplished with difficulty.

### Results

*Histology of resected masses*

Of the total of 73 patients 22% had histological evidence of residual tumour, 44% differentiated (mature) teratoma and 34% fibrosis and/or necrosis.

*Histology of primary tumour in relation to histology of restricted masses after chemotherapy (Table I)*

The majority of patients (75%) who showed differentiated teratoma in residual masses had MTI primary tumours. The converse was true in the group with fibrosis and necrosis where 60% had MTU primary tumours.

**Table I** Histology of masses resected after chemotherapy for advanced testicular non-seminoma in relation to the histology of the primary tumour (The Royal Marsden Hospital, 1976–1982)

| *Histology of of resected tissues after chemotherapy* | *Histology of primary tumour: % distribution* | | | |
|---|---|---|---|---|
| | *MTI* | *MTU* | *MTT* | *Sem AFP+* |
| Malignant tissue (16)[a] | 56 | 38 | — | 6 |
| Differentiated teratoma (32) | 75 | 19 | 6 | — |
| Fibrosis/necrosis (25) | 24 | 60 | 12 | 4 |

[a]No. of patients.

*Histological evidence of residual malignancy*

Of a total of 16 patients in this group only 7 (44%) are alive and disease-free (Table II). Of 4 men who did not receive chemotherapy after surgery only one is alive compared with 6/12 patients who were treated. As discussed below a raised serum marker level at the time of surgery was an adverse

**Table II** Outcome of patients with residual malignant tissue in masses resected after chemotherapy for non-seminoma: influence of post-surgical chemotherapy (The Royal Marsden Hospital, 1976–1982)

| *Post-surgery chemotherapy* | *No. of patients* | *Disease-free* | *Alive with disease* | *Dead of tumour* |
|---|---|---|---|---|
| Yes | 12 | 6 (50%) | 3 | 3 |
| No | 4 | 1 (25%) | 0 | 3 |
| Total | 16 | 7 (44%)[a] | 3 (19%) | 6 (38%) |

[a]Observation time since surgery: 6–78 months (median 23 months).

prognostic feature with only 1/9 patients alive and disease-free (see Table VI and Figure 3).

*Fibrosis and necrosis in residual masses*

As shown in Table III of 25 patients in this group 21 (84%) are alive and disease-free. Three patients had only part of their residual disease excised and all 3 relapsed. There was one post-operative death from secondary haemorrhage after abdominal surgery. Thus, none of the 21 patients who had residual masses completely excised and who are available for study have relapsed. Only one patient had a raised marker at the time of surgery (see below) and he relapsed having had an incomplete excision of residual disease.

**Table III** Resected masses after chemotherapy for advanced testicular non-seminoma: outcome after surgery of patients with fibrosis and/or necrosis (The Royal Marsden Hospital, 1976–1982)

| *Total patients* | *Continuously disease-free* | *Alive with disease* | *Dead of tumour* | *Post-operative death* |
|---|---|---|---|---|
| 25[c] | 21 (84%) | 2[b] | 1[a] | 1 |

[a]Incomplete surgery subsequent biopsy showed MTU.
[b]One patient had incomplete surgery and has residual static disease present. The second has incomplete surgery and has relapsed.
[c]Observation time 8–87 months (median 40 months).

**Table IV** Non-seminomatous germ-cell testicular tumours: outcome of patients with mature (differentiated) teratoma in masses resected after chemotherapy in relation to completeness of surgery (The Royal Marsden Hospital 1976–1982)

| *Extent of surgery* | *Total patients* | *Continuously disease-free* | *Relapsed* |
|---|---|---|---|
| Complete resection | 23 | 22 (96%) | 1 (4%) |
| Resected with difficulty | 5 | 4 (80%) | 1 (20%) |
| Incomplete | 4 | 1 (25%) | 3 (75%) |
| Total | 32[a] | 27 (84%) | 5 (16%) |

[a]Observation time 13–68 months (median 37 months).

*Differentiated (mature teratoma) in resected masses (Table IV)*

The most commonly reported tissues present in masses characterized as differentiated teratoma were squamous, respiratory or columnar epithelium, smooth muscle, cartilage, neural tissue, adipose and connective tissue. As discussed below cystic change was very common.

Although the overall prognosis of patients who show differentiated teratoma in resected masses is good, with 84% surviving disease-free (observation time 13–68 months, median 37 months), relapses may occur with risk being related to the completeness or otherwise of surgery as shown in Table IV. Thus whereas only 1/23 patients in whom a complete excision had been performed relapsed, 4/9 men where excision had either been accomplished with difficulty or been incomplete subsequently relapsed. Of the latter group one has died of tumour (27 months after surgery), two are alive with active disease (11 & 41 months) and one is alive with static disease (56 months). The patient who relapsed after an apparently complete excision showed raised markers as the only evidence of relapse and is disease-free after further chemotherapy at 36 months.

As shown in Table V cystic change was documented in 88% of masses showing

**Table V** Differentiated teratoma (TD) in masses resected after chemotherapy for non-seminomatous germ-cell testicular tumours: cystic change and enlargement during treatment (The Royal Marsden Hospital, 1976–1982)

| *Total patients with TD in surgical specimen* | *Cystic change present* | *Increase in volume of mass observed during chemotherapy* | *Appearance of mass* de novo *during chemotherapy* |
|---|---|---|---|
| 32 | 28 (88%) | 10 (31%) | 1 (3%) |

differentiated teratoma. Almost one-third of patients showed an increase in size of mass during chemotherapy and in one patient a cystic mass appeared although clinical staging had failed to reveal evidence of tumour at that site prior to chemotherapy. Enlargement of cystic masses during or after chemotherapy may produce symptoms and necessitate early surgery. In one patient in the present series bilateral ureteric obstruction occurred and it was necessary to drain cyst fluid by percutaneous needle aspiration.

Figure 1 shows the growth pattern in two pulmonary metastases in the patient designated as having static disease after the excision (with difficulty) of a retroperitoneal mass of differentiated teratoma. Numerous bilateral lung metastases appeared within one month of surgery the patient never having shown evidence of lung disease previously. Following initially rapid tumour growth most metastases have remained static, some showing very slow expansion over several years.

*Serum markers, surgical pathology and subsequent outcome*

Figure 2 shows serum marker levels prior to chemotherapy in relation to the histology of resected masses. There is no obvious difference in the distribution of patients with low and high marker levels in the three histological subgroups.

Figure 3 shows pre-surgical marker status in relation to eventual outcome in patients with histological evidence of residual malignancy in the resected specimen. As shown there is a high risk of relapse in patients with raised markers. In patients who were marker positive immediately before surgery the prognosis is poor even when post-surgical chemotherapy is given (Table VI). However

**Figure 1** Rapid appearance and subsequent growth pattern of pulmonary metastases resection of a residual abdominal mass showing mature teratoma after chemotherapy for Stage IIC testicular non-seminoma.

**Table VI** Patients with residual malignant tissue in masses resected after chemotherapy for advanced testicular non-seminoma: serum marker status and subsequent chemotherapy (The Royal Marsden Hospital, 1976–1982)

| *Serum marker status at time of surgery* | *Post-surgical chemotherapy* | *Number of patients* | *Disease-free* | *Alive with disease* | *Dead of tumour* |
|---|---|---|---|---|---|
| Marker positive | YES | 6 | 1 | 2 | 3 |
| patients (9)[a] | NO | 3 | — | — | 3 |
| Marker negative | YES | 6 | 5 | 1 | |
| patients (7) | NO | 1 | 1 | | |

[a]No. of patients ( ).

**Figure 2** Surgery after chemotherapy for advanced testicular non-seminoma: Surgical pathology in relation to pre-chemotherapy serum marker status.

**Figure 3** Post-chemotherapy excision of residual masses in testicular non-seminoma: influence of serum marker status at time of surgery on disease outcome in patients with evidence of residual malignancy in resected tissue.

in most cases surgery was performed in the marker positive patient because the possibilities of first line chemotherapy had been exhausted and only second line chemotherapy was possible after resection of the residual mass.

In the group with differentiated teratoma in the surgical specimen only one patient had raised serum markers (AFP 18 ng $ml^{-1}$ and HCG 14 iu $l^{-1}$) prior to surgery and this patient relapsed and is receiving further chemotherapy.

In the fibrosis/necrosis group only one man had a raised marker prior to surgery (HCG 16 iu $l^{-1}$) and he relapsed. However surgery was incomplete and at a second exploration residual MTU was confirmed.

Of the total of 17 relapsed patients abdominal recurrence occurred in seven (all with prior C disease), lungs in three (only one of whom had had lung metastases initially), lungs and abdomen in three (only one of whom had lung metastases initially), elevation of serum markers in one and extradural cord compression in one patient.

### Discussion

The present results confirm that the patients most at risk from relapse following resection of masses after chemotherapy for testicular cancer are those in whom there is histological evidence of residual malignancy in the surgical specimen, Such patients have a poor prognosis unless further chemotherapy is given. Marker status at the time of surgery, completeness or otherwise of resection and post surgical chemotherapy all influence the survival prospects of the patient. Bracken *et al.* (1983) reported that 9/17 patients who had residual malignant tissue resected and who were marker negative at the time of surgery were salvaged by further chemotherapy ± surgery. Vugrin *et al.* (1981) reported that of 11 marker negative patients who had complete resection of a residual mass showing histological evidence of malignancy nine were disease-free 5–33 months after surgery. The need for further chemotherapy after resection of active tumour is illustrated by the experience of Einhorn *et al.* (1981) who found that only 2/22 patients with resected carcinoma were continuously disease-free after surgery.

In patients with histological evidence of residual malignancy, those with raised serum markers at the time of surgery (that is after chemotherapy), even if there is only a slight elevation of one marker, are most at risk of relapse although if a complete resection can be achieved and there is scope for giving further effective chemotherapy the patient may be salvaged. Patients who are marker negative and who show focal residual malignant tissue within the surgical specimen have fared best, although even in this group further chemotherapy appears necessary. It is difficult from limited data to dissociate the diagnostic and therapeutic roles of surgery in patients with evidence of residual malignancy. However, the observation that the patients most at risk are those who do not have a complete resection, suggest that surgery contributes to cure by removing all macroscopically evident tumour. Support for the therapeutic contribution of surgery also comes from our limited historical experience where long term disease-free survival was achieved in patients from whom masses were excised after ineffective chemotherapy ± radiotherapy prior to 1975 (Hendry *et al.*, 1980).

The histological appearances of residual masses may be heterogeneous with an admixture of fibrosis, mature teratoma and residual malignant tissue. Thus, resection of part of a residual mass or only some of the metastases remaining after chemotherapy may predispose to relapse even in marker negative patients who do not have elevated serum markers after chemotherapy and prior to surgery and who show the histological appearances of fibrosis with necrosis or mature teratoma in the surgical specimen. Donohue *et al.* (1982) reporting on 51 patients who came to surgery after chemotherapy found that 16 (31%) showed fibrosis with necrosis, 16 (31%) mature teratoma and 19 (37%) residual cancer. In the fibrosis/necrosis group 2/16 patients who did not have complete resection subsequently relapsed. In a more recent report from the same group, of 54 patients who had teratomatous masses excised after chemotherapy (16 (30%) relapsed (Loehrer *et al.*, 1983). This included 41 patients with mature teratoma of whom 11 (29%) relapsed and 13 patients described as showing the appearances of immature teratoma of whom 5 (38%) had relapsed. A major factor predisposing to relapse was the completeness of surgical resection.

In some patients the subsequent spread pattern after surgery, when the histology is positive, suggests that haematogenous spread may occur at the time of resection. In patients with Stage II disease, in whom it is suspected that there may be residual malignancy present within the abdominal mass and in whom there is little in the way of chemotherapy reserve, the possible contribution of involved field irradiation should not be ignored (Duchesne & Peckham, 1984).

The presence of raised serum markers prior to surgery and after chemotherapy is associated with the histological findings of residual malignancy. Conversely negative markers do not predict the absence of a positive histology. This Vugrin *et al.* (1981) reporting on 47 patients operated upon after chemotherapy found that 8/9 marker positive patients had residual cancer whereas of 38 marker negative patients 11 (29%) had malignant tissue, 9 (24%) mature teratoma and 18 (47%) fibrosis/necrosis. Bracken *et al.* (1983) submitted 60 patients, all of whom had normal serum markers, to surgery after chemotherapy. Of this group 22 were judged to be in complete clinical remission after chemotherapy yet 5 (23%) had residual malignancy, 3 (14%) mature teratoma and 14 (64%) fibrosis/necrosis. In 38 marker negative patients with residual masses, 12 (32%) showed active tumour and 11 (29%) mature teratoma.

In patients with fibrosis and necrosis in the histological specimen the prognosis is good, only three failures have been seen, all in patients in whom there was incomplete excision of all disease and where relapse appeared in unresected tumour masses. These observations underline the fact that the histology of resected tissue in one site does not necessarily reflect that at other sites and that if possible an attempt should be made to excise all residual tumour after chemotherapy.

Perhaps the most fascinating group are those exhibiting histological evidence of differentiated teratoma. Differentiated teratoma may contain connective tissue, cartilage, smooth muscle, bone, nervous tissue, mucus secreting epithelium, squamous epithelium, ciliated epithelium and fat. Brodner *et al.* (1980) have described endocrine cells in 11/53 testicular teratomas associated with gastrointestinal epithelium. Most frequent were enterochromaffin cells but somatostatin, glucogen and pancreatic polypeptide immunoreactive cells were also identified. Bosman & Louwerens (1981) have also reported intestinal types of APUD cells in testicular teratoma.

In this series differentiated teratoma constituted 44% of patients who came to surgery and the overall prognosis is good. However, as with the previous two groups the completeness otherwise of surgery exerts an influence on subsequent outcome. Patients who have had a complete excision of residual masses and in whom there is only evidence of mature teratoma, have an excellent prognosis and no further treatment is required. The data are more limited for patients in whom surgery was difficult because of densely adherent tumour or where there was incomplete removal of residual disease. In both categories, however, the patient is at higher risk of relapse. In those patients where there has been resection only of part of the disease process, a subsequent attempt should be made to resect masses at all sites.

In practical terms patients with cystic differentiated teratoma may present a formidable problem in management, since surgery may be required for bulky cervical disease, intra-thoracic disease and masses in the abdomen and pelvis. Enlargement of masses may in itself be an indication for surgery because of the risk of airways compression, ureteric obstruction, etc. In such patients chemotherapy may be interrupted and the inter-digitation of systemic treatment and surgery may be intricate. Enlargement of cystic masses may be attributed to tumour growth and failure of chemotherapy if the evolution of expanding cystic differentiated teratoma is not taken into consideration (Logothetis *et al.*, 1982; Carr *et al.*, 1981). In some patients teratoma cyst fluid may contain high levels of AFP and/or HCG and this may be a guide to whether residual malignancy is present or not, and if so, which mass should be tackled as a surgical priority.

The phenomenon of differentiation in testicular malignancy is of great interest. Regression with spontaneous maturation of primary testicular tumours is a rare but well documented phenomenon. Azzopardi *et al.* (1961) reporting 17 patients who died of widespread germ-cell malignancy in whom no testicular primary was clinically evident found intratesticular cystic lesions in eight cases. This syndrome is often associated with trophoblastic malignancy and rapidly progressive disease carrying a poor prognosis (Powell *et al.*, 1983). The phenomenon of change towards a more benign state in metastases from testicular germ-cell tumours has long been recognised (Smithers, 1969). Numerous reports describe differentiated teratoma metastases excised after chemotherapy and/or radiotherapy (Duari, 1967; Willis & Hajdu, 1973; Merrin *et al.*, 1975; Stechmiller *et al.*, 1976; Hong *et al.*, 1977; Hendry *et al.*, 1980; Einhorn *et al.*, 1981; Vugrin *et al.*, 1981; Stahel *et al.*, 1982; Bracken *et al.*, 1983; Callery *et al.*, 1983; Oosterhuis *et al.*, 1983; van Dongen *et al.*, 1983). Whereas prior to the recent developments in chemotherapy this evolution towards morphological differentiation was uncommon it is now a well recognised feature.

An intriguing question is whether differentiation is promoted by treatment or whether chemotherapy eradicates undifferentiated elements leaving pre-existing differentiated structures *in situ*. Oosterhuis *et al.* (1982) have used mouse teratocarcinoma as a model for human testicular cancer to investigate the effects of *cis*-platin on differentiation and concluded that chemotherapy eradicates undifferentiated tumour leaving differentiated elements rather than promoting differentiation.

In the present series 75% of patients in whom differentiated masses were excised had intermediate malignant teratoma primary tumours, that is with evidence of differentiation present. As shown in Figure 4 radical lymph node dissection data from the prechemotherapy era indicate that the presence of differentiated elements in the primary tumour predisposes to some degree of differentiation in metastases. Even with earlier forms of therapy

**Figure 4** Histology of resected abdominal nodes in relation to histology of primary testicular tumour (Data taken from Ray *et al.*, 1974). (□) Primary tumour: non-seminoma with no differentiated elements (total 74). (▨) Primary tumour: non-seminoma with differentiated elements (total 46).

(radiotherapy and single agent chemotherapy) completely differentiated masses were encountered. This in our own experience of 13 (highly selected) patients operated upon between 1968 and 1976 for bulky residual disease four (30%) had fully differentiated residua (Hendry *et al.*, 1980). Recent data for Oosterhuis *et al.* (1983) demonstrate that there has been a progressive increase in the proportion of patients showing differentiated teratoma metastases as chemotherapy has become more effective; this increase is largely confined to patients who have differentiated elements in the primary tumour.

Induction of complete differentiation leading to fully mature end cells has obvious attraction as a therapeutic manoeuvre in germ-cell malignancy. The question arises as to whether there is evidence that the chemotherapy that has proved so effective in curing this once lethal disease acts at least in part by inducing differentiation. On presently available evidence it is not possible to exclude the possibility that differentiation is treatment-induced although the data can equally well be interpreted to indicate that chemotherapy has eradicated undifferentiated tumour leaving pre-existing differentiated elements *in situ*.

### References

AZZOPARDI, J.G., MOSTOFI, F.K. & THEISS, E.A. (1961). Lesions of testes observed in certain patients with widespread choriocarcinoma and related tumours. *Am. J. Pathol.*, **38**, 207.

BOSMAN, F.T. & LOUWERENS, J.-W.K. (1981). APUD cells in teratomas. *Am. J. Pathol.*, **104**, 174.

BRACKEN, B.R., JOHNSON, D.E., FRAZIER, O.H., LOGOTHETIS, C.J., TRINDADE, A. & SAMUELS, M.L. (1983). The role of surgery following chemotherapy in Stage III germ cell neoplasms. *J. Urol.*, **129**, 39.

BRODNER, O.G., GRUBE, D., HELMSTAEDTER, U., KREIENBRINK, M.E., WURSTER, K. & FORSSMANN, N.G. (1980). Endocrine GEP-Cells in primary testicular teratoma. *Virchows Arch* [*Pathol. Anat.*], **388**, 251.

CALLERY, C.D., HOLMES, E.C., VERNON, S., HUTH, J., COULSON, W.F. & SKINNER, D.G. (1983). Resection of pulmonary metastases from non-seminomatous testicular tumours. *Cancer*, **51**, 1152.

CARR, B.I., GILCHRIST, K.W. & CARBONE, P.P. (1981). The variable transformation in metastases from testicular germ cell tumours: the need for selective biopsy. *J. Urol.*, **126**, 52.

DONOHUE, J.P., ROTH, L.M., ROWLAND, R.G., ZACHARY, J.M., EINHORN, L.H. & WILLIAMS, S.G. (1982). Cytoreductive surgery for metastatic testis cancer: Tissue analysis of retroperitoneal masses after chemotherapy. *J. Urol.*, **127**, 1111.

DUARI, M. (1967). A primary malignant testicular tumour with unusual metastases. *Br. J. Clin. Pract.*, **21**, 195.

DUCHESNE, G. & PECKHAM, M.J. (1984). Chemotherapy and radiotherapy in advanced testicular non-seminoma (2) Results of treatment. *Radiother. Oncol.*, **1**, 207.

EINHORN, L.H. & DONOHUE, J. (1977). Cis-diamminedichloroplatinum, vinblastine and bleomycin combination chemotherapy in disseminated testicular cancer. *Ann. Intern. Med.*, **87**, 293.

EINHORN, L.H., WILLIAMS, S.D., MANDELBAUM, I. & DONOHUE, J.P. (1981). Surgical resection in disseminated testicular cancer following chemotherapeutic cytoreduction. *Cancer*, **48**, 904.

HENDRY, W.F., BARRETT, A., McELWAIN, T.J., WALLACE, D. & PECKHAM, M.J. (1980). The role of surgery in the combined management of metastases from malignant teratoma of the testis. *Br. J. Urol.*, **52**, 38.

HONG, W.K., WITTES, R.E., HAJDU, S.T., CVITKOVIC, E., WHITMORE, W.F. & GOLBEY, R.B. (1977). The evolution of mature teratoma from malignant testicular tumours. *Cancer*, **40**, 2987.

LOEHRER, P.J., WILLIAMS, S.D., CLARK, S.A. & 0 others. (1983). Teratoma following chemotherapy for non-seminomatous germ cell tumour: A clinicopathologic correlation. *Proc. Am. Soc. Clin. Oncol.*, **2**, 139.

LOGOTHETIS, C.J., SAMUELS, M.L., TRINDALE, A. & JOHNSON, D.E. (1982). The growing teratoma syndrome. *Cancer*, **50**, 1629.

MERRIN, C., BAUMGARTNER, G. & WAJSMAN, Z. (1975). Benign transformation of testicular carcinoma by chemotherapy. *Lancet*, **i**, 43.

OOSTERHUIS, I.W., SUURMEYER, A.J.H., SLEYFER, D., KOOPS, H.S., OLDHOFF, J. & FLEUREN, G. (1983). Effects of multiple-drug chemotherapy (cis-diammine-dichloroplatinum, bleomycin and vinblastine) on the maturation of retroperitoneal lymph node metastases of non-seminomatous germ cell tumours of the testis. *Cancer*, **51**, 408.

OOSTERHUIS, J.W., FOX, N. & DAMJANOV, I. (1982). Maturation of mouse teratocarcinoma treated with cis-diammine-dichloroplatinum (CDDP). *Proc. Am. Assoc. Cancer Res.*, **23**, 225.

PECKHAM, M.J. (1981). Testicular tumours: Investigation and staging: General aspects and staging classification. In: *The Management of Testicular Tumours*. (Ed. Peckham), London: Edward Arnold Ltd., p. 89.

PECKHAM, M.J., BARRETT, A., LIEW, K.H. & 5 others. (1983). The treatment of metastatic germ-cell testicular tumours with bleomycin, etoposide and cis-platin (BEP). *Br. J. Cancer*, **47**, 613.

PECKHAM, M.J., BARRETT, A., McELWAIN, T.J., HENDRY, W.F. & RAGHAVEN, D. (1981). Non-seminoma germ cell tumours (malignant teratoma) of the testis. Results of treatment and an analysis of prognostic factors. *Br. J. Urol.*, **53**, 162.

POWELL, S., HENDRY, W.F. & PECKHAM, M.J. (1983). Occult germ-cell testicular tumours. *Br. J. Urol.*, **55**, 440.

RAY, B., HAJDU, S.I. & WHITMORE, W.F. (1974). Distribution of retroperitoneal lymph node metastases in testicular germ cell tumors, *Cancer*, **33**, 340–347.

SAMUELS, M.L., LANZOTTI, V.J., HOLOYE, P.Y., BOYLE, L.E., SMITH, T.L. & JOHNSON, D.E. (1976). Combination chemotherapy in germinal cell tumors. *Cancer Treat. Rev.*, **3**, 185.

SMITHERS, D.W. (1969). Maturation in human tumours. *Lancet*, **ii**, 949.

STAHEL, R.A., VON HOCHSTETTER, A.R., LARGIADER, F., SCHMUCKI, O. & HONEGGER, H.P. (1982). Surgical resection of residual tumor after chemotherapy in non-seminomatous testicular cancer. *Eur. J. Cancer Clin. Oncol.*, **18**, 1259.

STECHMILLER, B., WIERNIK, P.H., SHIN, M. & SATTERFIELD, J. (1976). Metastatic teratocarcinoma following chemotherapy. Maturation to a mass pathologically indistinguishable from a mediastinal enteric cyst. *Chest*, **69**, 697.

VAN DONGEN, J.A., HUININK, TEN BOKKEL, W.W., VAN COEVORDEN, S.J., DELEMARRE, J.F.M. & VAN HOESEL, Q.G.C.M. (1983). The role of surgery in advanced testicular cancer. *Neth. J. Surg.*, **35**, 129.

VUGRIN, D., WHITMORE, W.F., SOGANI, P.C., BAINS, M., HERR, H.W. & GOLBEY, R.B. (1981). Combined chemotherapy and surgery in treatment of advanced germ cell tumours. *Cancer*, **47**, 2228.

WILLIS, G.W. & HAJDU, S.I. (1973). Histologically benign teratoid metastasis of testicular embryonal carcinoma. *Am. J. Clin. Pathol.*, **59**, 338.